# Contrasting Relativism, Absolutism and Pragmatism for Utility in Healthcare Ethics. Revisiting Drummond's Article on Relativism

**DOI:** 10.1111/nup.70041

**Published:** 2025-09-22

**Authors:** Pamela J. Grace

**Affiliations:** ^1^ Boston College Williman F. Connell School of Nursing Chestnut Hill Massachusetts USA

## Abstract

In this manuscript I revisit the late John Drummond's article on Relativism published in Nursing Philosophy (2005, volume 4). His was a very carefully developed look at relativism and its implications for healthcare work. Using John's account as a springboard, I contrast the philosophical approaches to knowledge acquisition and moral action of absolutism, relativism and pragmatism. I argue that pragmatism has more utility for ethical decision‐making in healthcare than either absolutist or relativist approaches by permitting the conceptualization of probable good actions for individual patients, and evaluating the rigor of underlying data‐gathering. Pragmatist methods also allow for evaluating the consequences of actions in terms of how well they achieved their goals, and accounting for this knowledge as pertinent when planning future actions. Because each healthcare profession has goals related to patient care, their practitioners need to be able to figure out how to meet those goals even in the face of obstacles. While restricted in their applicability, insights from both Immanuel Kant's moral absolutism and moderate moral relativism are useful in widening the scope of considerations and provide insights into the human condition. Pragmatism does not rule out this possibility unlike relativist and absolutist views which taken alone are mutually exclusive.

## 1 Introduction

The late John S. Drummond (d. 2014), author of the article I have chosen to discuss in this special edition, epitomizes McCullough's assertion. His writings demonstrate incisiveness and clarity. I remember him well for his humour, gentle personality, and clarity of thinking. A challenge from John in response to arguments he found lacking was always presented kindly and in the spirit of interested engagement. A founding member of the International Philosophy of Nursing Society (IPONS) and a regular contributor to Nursing Philosophy, his articles provide lucidity about, and insight into, the critical role of philosophy applied to nurses and for nursing purposes and thus perspectives on providing ‘good’ care. An eloquent tribute to John by Christine Ceci, Mary Ellen Purkis and Francine Wynn appeared in a 2017 edition of Nursing Philosophy (Ceci et al. 2017), and the reader is referred to this for more information about his life and work.

Here I revisit Drummond's discussion of *Relativism* (Drummond 2005), which I found very helpful in untangling the meanings of different versions, thus revealing the hidden implications especially related to nursing and healthcare practice as well as clinical ethics consultation. John is careful to note that his purpose is not to ‘set out to argue for or against relativism as a thesis. Rather (the purpose of his article) is to examine the term with a view to clarifying its uses and abuses at different levels and in different ways’ (p.267). In the end, this clarity is helpful for those interested in the ethics of nursing and healthcare because it goes some way to help us understand, and eventually refute, the argument that ‘if there are no moral facts, there can be no way to evaluate the ethical status of human actions.’ The problem is that if we think the absence of moral facts inevitably leads to *radical moral relativism*, then, what do we think we are talking about related to nursing and healthcare ethics? How do we evaluate the ethical status of actions? Logically, there is a middle ground and that is what John argues in his parsing out of the different meanings of relativism as discussed shortly. By logical I mean the philosophical sense that there is a chain of reasoning about a belief or proposition, the soundness of which can withstand challenges and counter examples (Klenk 1989). Eventually John reveals that the middle ground is a moderate version of relativism. While agreeing with John that the moderate moral relativist perspective, as he outlines it, permits critique of certain cultural practices thus the root causes of ill health for some, I argue that pragmatism may be a better bridge between absolutism and relativism. The pragmatist orientation is agnostic about whether there may be absolute truths, but also does not subscribe to the chaos that can result from relativist views on what is good in human action. Instead, it proposes that there are methods for both ‘(M)aking our ideas clear’ (Peirce 1878/1997, p. 26) and evaluating the soundness of ensuing actions (James 1907). For pragmatists, theories, logic and evidence are among the instruments one uses for conceptualizing which actions are likely to further a pre‐identified goal and for evaluating the results of those actions. Healthcare professional goals and responsibilities are inevitably ethical in nature because they are about providing for the human goods of safeguarding and restoring health, optimization of functioning, and relief of suffering (American Nurses Association [ANA] 2025; International Council of Nursing [ICN] 2021). However, barriers to good practice often arise and healthcare professionals are not always certain how to address these. I use John's clarifications as a foundation for arguing that pragmatism provides a perspective that is particularly useful for resolving ethical issues in healthcare and in fact, whether they call it pragmatism or not, it is the perspective that underlies the resolution of ethical conflicts contemporarily, in many countries.

But first, I provide a little background on the philosophical problem that, seemingly, pits the perspectives of absolutism and duty‐based approaches against various versions of relativism related to moral action. I am especially interested in how, as healthcare providers and clinical ethicists, we negotiate the fine line between rules that meet general needs and the potential for harmful consequences when rules/duties are applied indiscriminately in the care of those we serve.

## 2 Initial Clarifications: Moral Absolutism Versus Relativism

### 2.1 Moral Absolutism

Moral absolutism is the broad category under which theories of moral realism (Sayre‐McCord 2023), divinely given duties (require the existence of a higher power), or rationally conceptualized duties fall. Ruth Macklin (2011) captures the basic idea. ‘Absolutism is the view that there exist specific, exceptionless, moral rules that are valid for all people at all times and places, or that moral concepts have univocal and immutable meanings’ (p. 39). The various versions of absolutism are antithetical to relativism in any of its adumbrations. The idea behind moral absolutism is that rules about what is right or good exist and are derived from one of the following sources: Laws of human nature, pre‐determined edicts of a higher power—divinely revealed to human beings in some way, or rationally determined by human beings as described by Immanuel Kant (1785/1967). They have in common the idea that following rules or duties is what matters in actions towards other beings (and in some cases the environment), not the consequences of those actions. Duties that derive from the various forms of absolutism tend to be taken as normative or prescriptive. That is, actions considered from any of these perspectives are either valid, right, or good and ‘ought’ to be undertaken or are neutral or not permissible. Here I use Kant's theory that human beings have an inherent rational capacity to determine moral rules as an example of what absolutism commits us to, and why it is problematic in nursing and health care.

Kant's moral theory attempts to circumvent the idea that moral rules are divinely, or God given, by providing a detailed rational argument about the nature of human beings related to their ability to reason. In the *Critique of Pure Reason* (Kant 1781/1965) Kant provides a foundation for his later moral theory (Kant 1785/1969). Kant's Critique has been recognized as an attempt to bridge the gap between empiricist and rationalist traditions of philosophy related to knowledge acquisition. While noting that theories assigned to either tradition do not neatly nor entirely match their label, briefly, the Rationalists such as Descartes, Leibniz and Spinoza ‘shared a belief that it was possible by the use of reason, to gain a superior kind of knowledge to that derived from the senses’ (Cottingham 1988, p. 4). In contrast, the so labelled Empiricists such as Berkely, Locke, and Hume tended to emphasize the role of the senses and experience related to knowledge acquisition (Sperber 2015). Kant's often cited, ‘(T)houghts without content are empty, intuitions without concepts are blind’ (Kant 1781/1965, p. 93, A51/B76) illustrates his argument for the inextricable relationship between reason and experiences for knowledge production. As discussed next, both reason and senses play an important part in Kant's theory of moral reasoning and action, but it is the inherent human cognitive capacity to organize sensory input in a meaningful way, make moral judgements and choose to act on them that gives human beings moral worth as elaborated shortly.

### 2.2 Limitations of Moral Absolutism for Determining Moral Action

Importantly, using the example of Kant's theorizing, I am arguing that moral absolutist stances alone cannot help us to evaluate the ethical status of actions in healthcare and neither can relativist stances. Rather, it is insights from both stances, other perspectives such as feminist ethics, and the purposes of healthcare work (the ostensible reason for being of healthcare professions) that help us in ethical decision‐making in such settings. Later I discuss in more detail American Pragmatism as a way to resolve the problem that neither moral relativist nor absolutist perspectives are useful in resolving ethical problems in healthcare settings, although insights from these approaches do stimulate us to inquire into circumstances and context in more depth. According to absolutism, the rightness or wrongness of an action does not depend on how good or bad the consequences of the action are but rather on whether they follow a pre‐determined rule or commandment. Later I exemplify in what ways following rules without regard for consequences can work against professional goals and responsibilities. Likewise, I discuss why anticipating and evaluating likely consequences in a particular ethical conflict or situation, while important is insufficient without reference to context.

Kant's (1785/1967) moral theory is a form of absolutism and is presented briefly here as an example of what absolutism commits us to. In particular, while acknowledging that it provides us with important insights, it falls short of being able to provide direction in healthcare settings. I focus on Kant because many in healthcare are familiar with the role that the ethical principle of autonomy plays in patient care, underlying such concepts as rights to informed consent, and privacy and confidentiality among others. Especially in Western countries, the principle of autonomy as derived from Kant is often used uncritically to argue that people should be free to make their own choices, even when these may result in harm to them. In healthcare settings, the principle can be taken to mean we should not intervene when people are making bad choices about their treatments (Maier and Shibles 2011). However, healthcare work includes the obligation to help people get and understand the information they need to make a good decision for themselves. Moreover, we have learned so much more from research in cognitive psychology and other sources, since Kant, about the limited ability of human beings to make choices that are free of various influences (social, cultural, educational) (Doris and the Moral Psychology Research Group 2010; Kahneman 2011). Thus the principle of autonomy, as it is used to support decision‐making in healthcare practice is much more nuanced than that explicated by Kant. I return to this point shortly.

### 2.3 Sketching Kant's Moral Absolutist Argument

Kant argues that what constitutes a good action can be rationally determined and is morally binding. His theorizing revolves around the assertion that human beings are inherently rational beings. Moreover, a moral rule that results from the use of deliberative reasoning will be universally applicable. This ability is also what gives human beings dignity and moral worth. It is the idea that human beings have the potential to determine for themselves what constitutes an ethically or morally warranted action that gives human beings moral status. That is, they are ‘free’ to choose to act from the results of this deliberative reasoning. To say that people have equal moral status, however, is not the same as saying that people are equal in other respects. Opportunities, capabilities, and assets differ greatly among persons and Kant recognizes this. However, it is the inherent or a priori rational capacity (potential) to choose the right action along with volition or ‘will’ (freedom to choose an action) that underlies Kant's argument that persons should never be treated as an object that is used to further another's goals. They must be treated as important in themselves. However, it is beyond the purpose of the paper to describe in any length Kant's argument that human beings are 'ends in themselves'.

The human capacity for rational moral action as described by Kant has internal and external aspects. The internal aspects include the existence of an inherent receptive matrix (my term) that exists a priori in human minds. This attribute permits information from external sources and experiences to be organized into concepts and applied to a judgment about moral action. The matrix could be considered a sort of formula, although Kant's argument is much more intricate than sketched here and I suspect he would object to the label ‘formula’. Use of this inherent human attribute along with contextual information, he calls the Categorical Imperative (CI). The CI permits one to determine the permissibility of a given action. Initially the CI has no content as such, rather knowledge from prior experiences as well as sensory and environmental aspects of a situation (Kant 1781/1969) are used by an individual to populate the CI in a way that permits knowing the permissible or prohibited action in a given situation. Thus, the external aspects are not all ‘external’ in the sense of existing outside of the person but include knowledge from prior and current sensory experience and the a priori existence of a capacity for organizing information in a way that its moral status can be discerned. However, a third aspect is that of the volitional ability (will) to choose the right action, or not. This third aspect implies human freedom to make intentional choices, as discussed in more detail shortly. There are several versions of the CI formula that Kant considers equivalent. He uses the different versions to demonstrate separate aspects of his argument that each human being is of moral worth. Individual human beings must be respected because of their capacity to reason about the permissibility, obligatory, or forbidden nature of a proposed action (Grace and Milliken 2022; Grace and Uveges 2022). Later I exemplify in what ways this ‘duty‐based’ account may provide insights but is insufficient for ethical decision‐makig in healthcare practice.

For the current purposes of illustrating moral absolutism I will use the version of the CI that states, “(A)ct only according to that maxim by which you can at the same time will that it should become a universal law” (1785/1967, p. 339). Roughly restated, if on reflection you could agree that anyone in the same situation would be justified and indeed ought to act the same way that you are about to, then that is the morally defensible choice because it is the reasonable choice; and it is one's duty to complete (or refrain from completing) this action. Moreover, it answers the question, ‘Would we be willing to support such a rule if proposed by another person’? An example of this given by Kant himself is that one should never lie because lying is irrational. Rationally, if I can lie then anyone can and we would not be able to trust communication … while this might sound consequentialist it is not the consequences that matter so much as the fact that we can reason that this act is never ethically permitted regardless of individual consequences. However, whether we do act from the CI is dependent on the other essential internal aspect of moral action, a ‘good will’ that resides in human beings.

The good will is not good because of what it effects or accomplishes or because of its adequacy to achieve some proposed end: it is only good because of its willing, i.e., it is good of itself.

Kant (1785/1967, p.322)

This is the volitional part that allows the freedom and impetus to act on what has been determined as the moral action in a situation. Intentionality is part of the process. Indeed, action that results from the dictates of the CI in accord with the ‘good will’ is a duty; it is a rationally mandated action. It also implies that human beings have a ‘free will’ in the sense that they may choose to follow the CI or ignore it. As Kant argued, ‘(W)hen we observe ourselves in any transgression of a duty, we find that we do not actually will that our maxim (the CI) should become universal law’ (Kant 1785/1967, p. 341). In trying to do the right thing, we must act ‘from the duty’ exposed by the CI. Not doing so is irrational. The prior example that one has a duty not to lie supports this idea. Kant gives an example of a person who needs to borrow money, but although he knows he will not be able to pay it back promises he will. The CI cannot be used in defense because he could not ‘will’ this to be a universal law. If lying were permissible, we would not be able to trust what anyone says. This is both irrational and treats the other person as merely a means to the proposed debtor's ends (Kant 1785/1967, p. 340).

#### 2.3.1 Critiques of Kant's Theory

As usual in evaluating moral theories and their usefulness in human life, there are a variety of critiques. I will note just a few here that are pertinent to the idea that moral absolutism as per Kant cannot on its own help us resolve ethical problems in healthcare. The ethical principle of autonomy derived from Kant's exegesis has been useful in drawing our attention to the need for everyone to be treated as of moral worth, indeed it underlies the idea of human rights and in this sense has utility. If everyone is to be treated as having moral worth, then there should be ways (perhaps laws) that protect the individual's rights, for example, not to be harmed, enslaved and so on. However,

1. The theory itself is not attentive to the specifics of a situation. In healthcare, context matters, and ethical situations are complex (O'Neill 1998). How specific would we need to be in applying the CI to a situation. For example, an extremely independent patient in imminent sepsis is saying they want to leave the hospital against medical advice. However, they have not understood the dangers of this (to their life and therefore the threat to any future exercise of autonomy). How much detail would need to be provided to determine what any other healthcare provider (HCP) in the ‘same’ situation should do? Is the level of detail that would be needed possible to obtain?
2. Different directions may result from the use of CI in a situation. For example, telling the stark truth to someone (not lying) may conflict with not causing unnecessary harm.
3. Consequences matter in healthcare as healthcare professions exist to provide services related to human health, functioning and flourishing. We have responsibilities to try and provide the services needed for the person and anticipate and reduce likely harms as part of our professional goals.
4. Studies in cognitive science and moral psychology have called into question the ability of human beings to act totally rationally. We are influenced by things that we may have control over and others that we do not (Kahneman 2011). Estimates are that only a small percentage of brain processes (about 10%) are under conscious control (DeWall et al. 2008).

## 3 Drummond on Relativism

Unlike absolutism, relativism does not depend on ‘ideal’ or absolute moral facts. It is a set of views essentially asserting that what is right or wrong is relative to contexts and circumstances. It is not a new theoretical perspective; its roots go back to debates during the time of Plato. Nevertheless, coming to agreement about what relativism is and why, or whether it is important, remains difficult. For the interested reader, Baghramian and Carter (2022) provide a fine‐grained and detailed overview of the many forms of relativism and their historical origins, highlighting just how complex a concept it is. Drummond (2005) starts his exploration by noting that, ‘(W)hile relativism can often be precise in meaning, it is also at certain levels of abstraction, slippery, and open to both abuse and confusion’ (p. 267). We have all heard the comment that ‘my beliefs are as relevant as yours,’ or ‘relativism is a problem because it means anything goes and there is no way to determine what is better or best in a situation.’ It is this problem that Drummond tackles.

First, Drummond traces the logic behind relativism. This is critical because his discussion takes as understood that relativism necessarily relates one thing to another in evaluating the truth of a statement. He gives the example of ‘Jane is tall…relative to Sally.’ The truth of this statement lies in the measurable fact of Jane's height relative to Sally's height. Not all truths, though, depend on relationships. A ‘truth’ can just be an observable situation. For example, as I sit here in my dining room in New Hampshire, I can see that it is snowing outside. The truth of this is provable by my going outside and feeling the snow on my cheeks. This is not a relational truth; nevertheless, both situations are logically equivalent. Understanding this ‘helps us to ask the right questions’ (Drummond 2005, p. 268). The right questions, then, may be posed in response to the statement ‘relativism means anything goes.’ We can now ask, ‘what is this “it” that is all relative, and what is it about “it” that is relative? And second, relative to what’ (p.269)?

### 3.1 Main Types of Relativism

As noted earlier, there are several versions of relativism, and the characteristics and conclusions of each, as well as their relationships to each other need to be differentiated for clarity. Drummond describes three main types and their criteria and implications. These are cultural, moral and epistemological. In the end it is moral relativism that is in question when it comes to evaluating problems in patient care and what is needed to resolve them. However, cultural relativism is also important in nursing settings because we have come to accept that culture plays an important role in what people believe about themselves; thus, what they want for themselves depends to a certain extent on their culturally derived beliefs and values. Additionally, healthcare providers have their own culturally and experientially derived beliefs and values that can introduce bias and prejudice into their attitudes toward patients. Our role is to clarify, via our clinical knowledge, in what ways it may be possible to meet patient goals given apparently conflicting cultural beliefs and values.

#### 3.1.1 Cultural Relativism

Cultural relativism is based on the premise that much, if not all, of what people believe, value, or take as normative will be related to aspects of the culture in which they live, and that this may differ (and may be held to be better or worse) relative to other cultures.

(Drummond 2005, p. 268)

Drummond notes that two senses of cultural relativism tend to be prominent in literature. Modest cultural relativism refers to the more everyday meanings of the culture. This is the anthropological description of the ‘what is’ of different cultures and sometimes how beliefs and customs have come to be as well as who seems to fare better or worse in terms of opportunities to flourish or ill health. Understanding that differences among cultures exist permits a pathway for mutual respect, learning, and sometimes change. Modest cultural relativism might allow us to critique certain morally problematic customs both in our own culture and within other cultures. For example, some people living within the culture may be treated as if their beliefs and values do not matter, or are seen as of less standing or moral worth than others within the culture or are engaging in practices that are harmful but not understood as so. However, change is unlikely to happen without the realization of powerful members of the group that the bad effects of a custom or practice outweigh what they see as the benefits or find acceptable. An example of this might be infundibulation, also known as female genital mutilation (FGM), the various types of which can cause harm to women. Infundibulation, is still practiced in certain parts of the world (Gruenbaum 2006; World Health Organization [WHO] 2025). While the practice of FGM continues, changes are being made slowly as the health implications become better understood within the culture. It is likely that each culture has practices or norms that harm certain groups within the population. For example, an emphasis remains on extreme slenderness as a symbol of desirability in females in the US and elsewhere.

Strong cultural relativism, on the other hand, does not allow for critique of other cultures. Each culture is to be viewed through its own lens. As Drummond noted, ‘the modest cultural relativist seeks to stop short of moral or epistemological relativism’ (p. 268). In other words, we know that some beliefs, values, and customs within a culture can cause harm to persons or groups of persons and critiquing the justice of these is acceptable on the modest relativist perspective with the caution that the necessary changes while perhaps stimulated by empirical studies, nevertheless, are initiated within the culture.

The strong version of cultural relativism, as Drummond (2005) notes, however, does not allow for critique of unjust systems (i.e., some members of a given society are treated of less concern than others). The assertion is that there

is no objective point of reference for judging that the aspects and values of one culture (religion, economics, or gender relations for instance) are any better than in another culture.

(p.268)

It is to this idea that John objects from an ethical perspective. Relativism cannot commit us to ignoring injustice, a logical argument contra this exists. Especially, in healthcare we may be witness to persons suffering injustices of various sorts. To ignore recurring problems, such as chronic diseases that have progressed beyond easy management because of lack of access to healthcare is to not take our professional responsibilities seriously (Grace 2001). The problem is that both within and across cultures, groups of persons can be treated as less than fully human. Large swaths of a population may be discounted as being of no or less moral concern than others (e.g., slavery, patriarchal societies, poor access to basic social services that are available to others in the community). Research in cognitive psychology increasingly supports the multifactorial concept of human bias and its evolutionary role in protecting a group and advancing its concerns at the expense of others within, or outside of, the group (Wong and Vinsky 2021). This leads to the problem that human beings can be harmed without penalty if it serves the desires of a powerful group. Thus, strong or radical relativism allows injustices. John's next task is to show how moral and epistemological relativism are related to modest and strong cultural relativism and, for current purposes, how this relates to healthcare and other critical human services. By critical, I mean those services that help people to function and flourish. Windt (1989), notes that such professions as medicine, nursing, law have a ‘grinding, life‐or‐death sort of importance’ (p.7). Yet if strong moral relativism is accepted, there would be no way to hold professionals accountable for not fulfilling their goals or for allowing injustices in healthcare delivery systems to persist. This is where Drummond's analysis helps us to be clear about why moral relativism is not applicable to, or in, healthcare practice.

#### 3.1.2 Moral Relativism

John asserts that ‘(M)oral relativism rests on the premise that there would appear to be no objective standpoint from which moral issues can be securely judged in an epistemological sense’ (Drummond 2005, p. 269). Why not? Because two things are conflated. Morality is about what is good, not about what is true (epistemology). Moral action is about action that produces a ‘good’ or refrains from harming. The ‘good’ is in relation to something to be achieved, thus it is not ‘good’ in and of itself. The concept of ‘good’ itself is abstract and indefinable—not able to be analyzed. G.E. Moore (1903/1967) argued that good is indefinable in the same way that ‘yellow’ is indefinable. That is ‘yellow’ cannot be further broken down into component parts. We might say this is a yellow rose, and we can further define rose as a flower of a certain type, but we cannot go on to define yellow. For Moore, it is the same with the concept of good. We can define something as a good X, or a good feeling, and we might generally know what this means in relation to something else, for example, it is more perfect in its design or ability to function, but ‘good’ itself is not further definable without reference to, or modification by, context and/or purpose.

Determining a moral action, one aimed at producing a ‘good’ or minimizing a harm, depends on who will be the focus of the action, what is available (resources), what is known with reasonable certainty (pertinent scientific and contextual knowledge), and the beliefs, values and preferences of the person in question. Those involved in the decision‐making process also need to examine their own beliefs, values, and potential biases for their likelihood of interfering with completion of the moral action. Beliefs and values both tend to change with time, experience, and the freedom to think for oneself, highlighting their mutability when subjected to analyses. Moreover, advances in cognitive science have revealed the many biases that can distort thinking in a way that can harm others (Korteling et al. 2023). We may be influenced by cultural values, but we need not be directed by them as long as we stay mindful that our thinking can be distorted, and seek to question assumptions we are making. Drummond calls this the ‘modest’ or ‘flat’ version of moral relativism.

However, this leads us to question whether there are some values that persist across persons and eras. An example might be that people generally have an interest in surviving and not being unnecessarily harmed (in the absence of serious mental health problems). Additionally, the idea of universal human rights can be described secularly and logically, based on the argument that if anyone or any group can be treated as less morally worthy than others, for example, treated as an object, anyone of us at any time could fall into the group who is discounted. Thus, we all have an interest at least in the principle of justice as fairness (Rawls 1971), although how to adhere to and maintain this is less clear.

Drummond (2005) then goes on to discuss the strong version of moral relativism, which is basically ‘anything goes’ and argues that it is unlikely any philosopher would subscribe to this view. Certainly, in healthcare settings we cannot follow an ‘anything goes’ perspective because we have goals to meet and the knowledge and skills to meet these goals or at least understand when our knowledge and skills are inadequate. Those goals in a clinical setting involve another person whose ‘good’ relative to the context is in question. That person's values, beliefs and desires are part of the decision‐making process, even if they do not cohere with what we would want for ourselves. Finally, Drummond discusses epistemological relativism and its relationship to cultural and moral relativism.

#### 3.1.3 Epistemological Relativism Also Known as Cognitive Relativism

‘What is meant by all truth is relative’? Drummond poses this question (p. 270). This is the issue at stake in epistemological relativism. Epistemological relativists posit that whether absolute truths exist is unknowable because human beings lack the ability to be uninfluenced by their prior experiences, culture, and context. and context. We cannot take the stance of an ‘ideal observer’ (Firth 1952); one who is uninfluenced by contexts and circumstances, ‘capable of reacting in a manner which will determine by definition whether an ethical judgment is true or false’ (p. 321). Firth (1952) postulates that were an ideal observer able to exist, they would be omniscient (knowing everything), omnipercipient (perceiving everything), disinterested, dispassionate, consistent and normal. In other words, not human. But Drummond argues we do not need absolute truths to function. Truths can be contingent, but still somewhat trustworthy unless demonstrated to be otherwise. Elaborating on Drummond's take, with apologies for any liberties I have taken, some truths, as noted earlier, are not so much relative to something else, as they are observations in the moment, such as ‘it is raining, and I don't have an umbrella.’ For nursing practice and nursing knowledge development—which is why gaining philosophical clarity is critical—the view of truth as relative does not mean ‘anything goes’ but rather what can we rely on as a basis for action. Conversely, deontologic rules strictly followed can also be problematic. For example, honoring a person's autonomy, from a Kantian point of view, in healthcare settings can lead to the paradox that the person who has come seeking help for their health, functioning, or flourishing, chooses an option that can lead to harm. For example, a person may be unable to make a good decision for themselves (cognitive problems, delirium, etc.). A person with impending sepsis may refuse antibiotics in the belief that they are antipsychotics drugs and previously they had had a bad experience with these drugs. We would not be justified in simply going along with this decision. The person would likely die and thus no longer be able to exercise any sense of autonomy. We would try to work with them to see how what is being proposed is likely to further their preferences or life trajectory in so far as these are known. These are also our professional goals. A process of deliberation is required on the part of the patient, related to the choice to be made. The person must be able to take in information, process it considering their values and preferences, and describe how the proposed intervention is or is not likely to be in‐line with preferences for their future (Beauchamp and Childress 2019). This process in turn requires that we ‘experts’ provide necessary information in a way that the individual can understand. Next, I describe why I consider pragmatism to better capture ethical decision‐making in clinical situations.

## 4 Insights From American Pragmatism

American pragmatism as a philosophical perspective, neither relies on the idea that there are absolute moral truths, nor in the idea that there are can be no objective ways of evaluating the value of actions. The pragmatist view of truths related to action, is that there are contingent truths that can be fortified or dispelled with analysis, evidence and consequences. The American Pragmatist movement developed in response to societal changes. Most importantly the American Civil War (1861–1865) affected prominent thinkers. “The rigid certainties and inflexibility that led to the war reflected a particular mindset, one attached to certainties and an unwillingness to compromise” (Bacon 2012, p.3). Dewey (1925/1989) summarizes the general idea behind pragmatism, ‘knowledge is an affair of *making* sure, not of grasping antecedently given sureties’ (p. 128). It is in using what is taken to be at least tentatively known as able to accomplish a goal or solve a problem that validation or revision occurs. While pragmatism is conceptualized slightly differently by its theorists, there are common threads. C.S. Peirce (1839–1914, William James (1842–1910), John Dewey (1859–1952) were progenitors, and they are followed by more contemporary thinkers including Richard Rorty. Pragmatism resists the exclusionary dualisms of absolutism and radical relativism. It also applies in areas where modest moral relativism is not apt. As James argued

(G)rant an idea or belief to be true… what concrete difference will it being true make in anyone's actual life? How will the truth be realized? What experiences will be different from those that would obtain if the belief were false?

James (1997/1907, pp.113‐4)

Zhang et al. (2024) present a coherent explanation of pragmatism as providing a way to avoid the dichotomy of moral absolutism and moral relativism when needing to make a moral judgment and act upon it. They call this middle way, moral pragmatism. This idea of moral pragmatism tends to be the way that complex ethical issues and conflicts are addressed in ethics consultation currently. Ideally, it is also the way that everyday ethical practice proceeds, using clinical judgement (Haggerty and Grace 2008).

### 4.1 Moral Pragmatism and Ethical Decision‐Making in Healthcare

The goals of the various healthcare professions should serve as an anchor for decision‐making in practice. Professional goals are ethical goals as argued earlier. The use of evidence, knowledge, skills, and experiences are necessary to decide likely best actions for a patient and choose the one most likely to resolve the issue. Additionally in complex situations and conflicts, decision‐making heuristics are utilized to ensure broad consideration of pertinent factors. A synthesized heuristic for everyday nursing practice issues is available (Grace 2009). Another heuristic conceptualized by Jonsen et al. (2002) is one used in clinical ethics consultation and for complex conflicts. The authors aimed to provide ‘a structured approach for identifying, analyzing and resolving ethical issues in clinical ethics’ (2002, p.1). They propose a four topics model that incudes understanding the medical situation, patient preferences, quality of life considerations and contextual features (Milliken et al. 2022). Important in this process is exploration of patient goals and preferences, assumptions, fact‐finding, and pertinent evidence. Further, evaluation of the consequences of our decision‐making permits identification of whether our decision making achieved or failed to achieve what was intended and enables application of this learning to subsequent problems.

## 5 Conclusion

John Drummond provided a valuable service in clarifying the ideas behind various types of relativism and addressing their critiques. In terms of ethical decision‐making in nursing and healthcare settings, understanding the tenuousness of what initially presents as a truth, helps us explore the nuances of problem, be clear about what is going on, and act to meet the goals of care. However, both modest moral relativism and Kantian absolutism are limited in their ability to resolve both everyday ethical issues and complex ethical conflicts, for the reasons given above. Because healthcare professionals do have ethical responsibilities to provide the services which are their reason for existence, a middle ground is needed for solving individual patient care problems. The moral relativist approach might allow us to resolve a patient care situation, but we still need a way to evaluate how well that situation was resolved, and what the implications for the larger society might be. Kantian absolutism might focus on prioritizing the patient as being of moral worth and for whom a resolution is needed but does not tell us how to balance conflicting principles. Pragmatism provides the bridge between these two perspectives. It does so by permitting recognition that each practice issue or ethical conflict is novel in some respects, and requires an approach that can reconcile unknowns with existing knowledge to achieve a pre‐set goal—the good of the person in question.

One does not have to cling to the idea that there are absolute truths to do the right thing when the right thing is right relative to the goals of nursing practice (its raison d’être), the context of care, and the person at the center of a nurse's concern. When it comes to nursing practice, metaethical questions such as, “What is the meaning of ‘good’ or ‘right’ in a human living context?” is relative to the problem at hand and is almost always somewhat dependent on the subject's perspective about their quality of life, or a proxy decision‐maker's representation of what the subject would have wanted.

## Ethics Statement

This is a discussion paper no IRB review needed as no human protection issues.

## Conflicts of Interest

The author declares no conflicts of interest.

## Data Availability

As a discussion paper no data was drawn upon. All articles referenced are publicly available.
